# Nuclear Regulation of Wnt/β-Catenin Signaling: It’s a Complex Situation

**DOI:** 10.3390/genes11080886

**Published:** 2020-08-04

**Authors:** Christin C. Anthony, David J. Robbins, Yashi Ahmed, Ethan Lee

**Affiliations:** 1 Department of Cell & Developmental Biology, Vanderbilt University, Nashville, TN 37232, USA; christin.c.anthony@vanderbilt.edu; 2 Molecular Oncology Program, Division of Surgical Oncology, Dewitt Daughtry Family Department of Surgery, and Sylvester Comprehensive Cancer Center, Miller School of Medicine, University of Miami, Miami, FL 33136, USA; drobbins@med.miami.edu; 3 Department of Molecular and Systems Biology and the Norris Cotton Cancer Center, Geisel School of Medicine at Dartmouth College, Hanover, NH 03755, USA; yashi.ahmed@dartmouth.edu; 4 Vanderbilt Ingram Cancer Center, Vanderbilt University Medical Center, Nashville, TN 37232, USA

**Keywords:** Wnt/β-catenin signaling, nuclear regulation, TCF/LEF, TLE, Pygopus, BCL9, enhanceosome

## Abstract

Wnt signaling is an evolutionarily conserved metazoan cell communication pathway required for proper animal development. Of the myriad of signaling events that have been ascribed to cellular activation by Wnt ligands, the canonical Wnt/β-catenin pathway has been the most studied and best understood. Misregulation of Wnt/β-catenin signaling has been implicated in developmental defects in the embryo and major diseases in the adult. Despite the latter, no drugs that inhibit the Wnt/β-catenin pathway have been approved by the FDA. In this review, we explore the least understood step in the Wnt/β-catenin pathway—nuclear regulation of Wnt target gene transcription. We initially describe our current understanding of the importation of β-catenin into the nucleus. We then focus on the mechanism of action of the major nuclear proteins implicated in driving gene transcription. Finally, we explore the concept of a nuclear Wnt enhanceosome and propose a modified model that describes the necessary components for the transcription of Wnt target genes.

## 1. Introduction

Wnt signaling is an evolutionarily conserved cellular communication pathway that plays critical roles in development, tissue homeostasis, and, when misregulated, human disease [1]. Wnt ligands trigger discrete intracellular cascades, resulting in a plethora of distinct cellular responses. The best characterized branch of the Wnt pathway is the Wnt/β-catenin signaling pathway, and most attention has been focused on its plasma membrane and cytoplasmic regulation. In this review, we focus on its less well described nuclear regulation.

**Figure 1:**
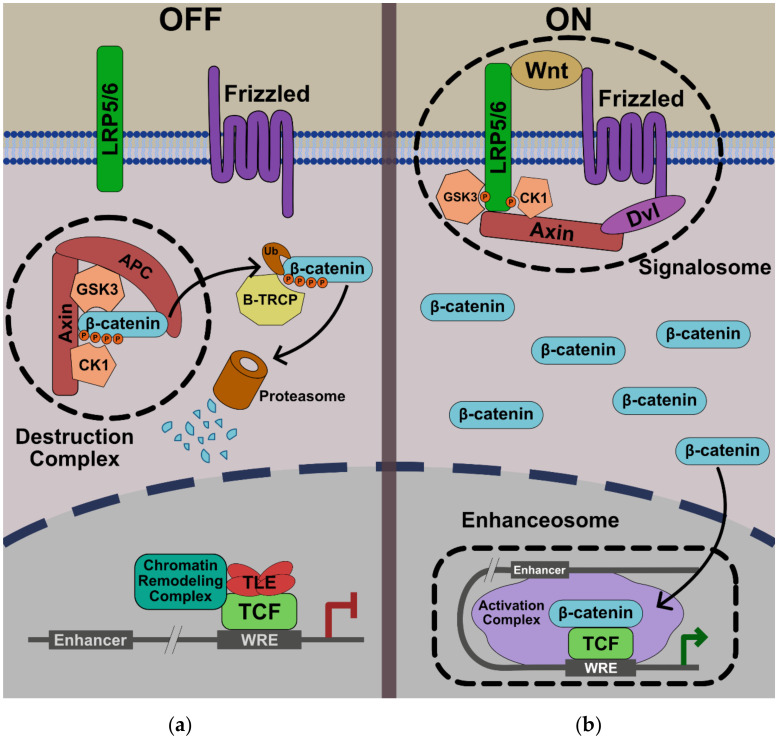
Model of the Wnt pathway. (**a**)—ln the absence of Wnt ligand, the β-catenin β destruction complex maintains low cytoplasmic levels of β-catenin. (**b**)—In the presence of Wnt ligand, the signalosome is assembled and the β-catenin degradation is disrupted. Entry of β-catenin into the nucleus promotes the formation of the enhanceosome to drive the transcription of Wnt target genes. See text for more details.

The current model of Wnt/β-catenin signaling includes the assembly of two distinct large protein complexes (Figure 1). In the absence of Wnt ligand, the transcriptional co-activator, β-catenin, is constitutively degraded by the cytoplasmic “destruction complex” (composed of the scaffolding proteins Adenomatous polyposis coli (APC) and Axin, and the protein kinases glycogen synthase kinase-3 (GSK3) and casein kinase 1α (CK1α) [2]. In this model, phosphorylation of β-catenin by CK1 at position serine 45 primes β-catenin for processive phosphorylation by GSK3 at nearby sites (Serine 33, Serine 37, and Threonine 41). Phosphorylated β-catenin is recognized by the β-Transducin Repeat Containing E3 Ubiquitin Protein Ligase (β-TRCP), which catalyzes its polyubiquitylation. Polyubiquitylated β-catenin, in turn, is degraded by the proteasome. In this way, cytoplasmic β-catenin levels remain low in the absence of Wnt.

Binding of Wnt ligands to the co-receptors Frizzled and LRP5/6 and the cytoplasmic protein Dishevelled (Dvl) induces a conformational change that is transmitted to the intracellular domain of Wnt receptors via a mechanism that remains poorly defined. The formation of a large oligomerized Frizzled-LRP5/6 complex or “signalosome” is thought to be critical for formation of an active receptor complex [3,4]. Phosphorylation and degradation of β-catenin within the destruction complex is ultimately blocked, β-catenin accumulates in the cytoplasm, and translocates to the nucleus. Once in the nucleus, β-catenin acts as a co-activator for T-cell factor/Lymphoid enhancer factor (TCF/LEF) on Wnt Response Elements (WREs) in target genes to transmit a Wnt transcriptional program. Recently, it has been proposed that this transmission of a Wnt signal in the nucleus via β-catenin-TCF/LEF occurs via a large molecular weight “enhanceosome” complex (Figure 1) [5].

## 2. Nuclear Transport of β-Catenin

The entry of β-catenin into the nucleus is a major signaling step in the Wnt pathway, although a consensus regarding its mechanism of action remains elusive. The FG repeats of nucleoporins normally block nuclear entry for proteins larger than 40 kDa [6]. In the classic nuclear transport model, nuclear localization sequence (NLS)-containing proteins bind to Importin-α, which is brought to the nuclear pore complex (NPC) and transported through the nuclear pore via its interaction with Importin-β. Once inside the nucleus, the protein cargo is released via the action of Ran GTPases [7].

β-catenin has no identifiable NLS [8], and numerous non-classic models for β-catenin nuclear import have been proposed. The 12 Armadillo repeats of β-catenin are similar to the HEAT repeats found on Importin-β family members, suggesting that β-catenin might directly interact with the NPC [7]. Based on the demonstration that nuclear transport occurs independently of FG-nucleoporins, and the fact that β-catenin transport does not compete with Importin-β, it has been proposed that β-catenin might be transported via a pathway distinct from that involving Importin-β [8]. More recent studies have identified several distinct regions of β-catenin (e.g., N-terminal, C-terminal, and Armadillo repeats 10–12) that can bind weakly to nucleoporins, suggesting that cumulative interactions may be required for nuclear import [6].

Various “piggyback” or “chaperone” models have also been proposed for the nuclear transport of β-catenin. One candidate is APC, which binds tightly to β-catenin and is localized to both the nucleus and cytoplasm [9]. A similar model has been proposed in which β-catenin is anchored to the nucleus via its interaction with B-Cell CLL/Lymphoma 9 (BCL9) [10]. Interaction between β-catenin and BCL9 has been proposed to be mediated by the phosphorylation of β-catenin at Tyrosine 142 [11]. Although the most obvious piggyback candidate for β-catenin is LEF1, nuclear entry of β-catenin has been shown to occur independently of this transcriptional factor [12].

Recently, the guanine nucleotide exchange factor, RAPGEF5, and its associated Rap1a/b GTPases have been shown to play a role in β-catenin nuclear import. This new pathway has been proposed to act in a manner that parallels the Ran/Importin-β transport system [13]. In addition, a Kinesin 2/IFT-A complex has also been shown to promote nuclear translocation of β-catenin in *Drosophila* and mice [14]. It remains to be determined whether these various models of β-catenin nuclear import reflect redundant and/or tissue-specific mechanisms. Finally, post-translation modification of β-catenin by O-GlcNAcylation stimulates its nuclear export, although the mechanism by which this occurs is unknown [15].

## 3. Nuclear Mediators of Wnt Signaling

Several proteins that regulate the transcription of Wnt target genes have been identified. In this section, we discuss the best characterized of these proteins: β-catenin, T-cell factor/Lymphoid enhancer factor (TCF/LEF), Groucho/Transducing-Like Enhancer (Gro/TLE), Pygopus (Pygo), and B-cell CLL/lymphoma 9 (BCL9). Some of these proteins, however, may not be absolutely required for Wnt signaling and instead may only function in certain contexts. We also discuss β-catenin-mediated transcription of genes that involves non-TCF/LEF partners, but are, nevertheless, dependent on Wnt signaling.

### 3.1. β-Catenin

β-catenin is often referred to as the master regulator of Wnt signaling because it is the transcriptional switch that shifts TCF/LEF from repressor to activator. The general role of β-catenin is to facilitate 1) the recruitment of the Mediator complex essential for RNA Polymerase II-mediated transcription [16] and 2) the binding of histone acetyltransferases to open chromatin, thereby promoting the recruitment of general transcription factors to target gene promoters [17].

**Figure 2:**
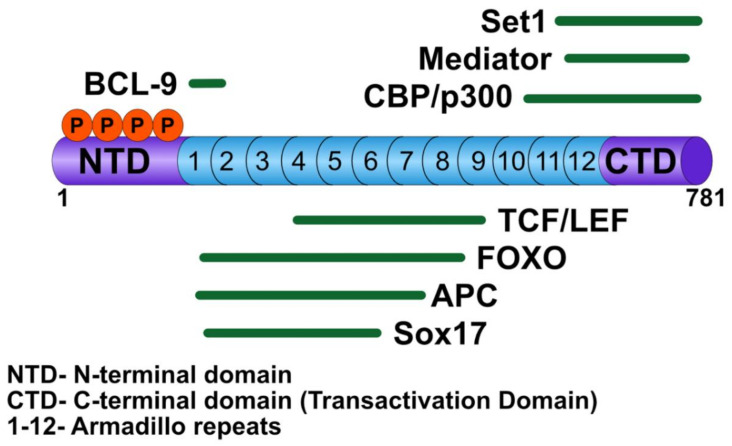
Cartoon of β-catenin and its nuclear interacting partners.

β-catenin has multiple nuclear binding partners that play critical roles in the activation of Wnt target genes, including TCF/LEF transcription factors [18], BCL9/BCL9-2 [19], CREB binding protein (CBP)/p300 [20], and SET domain-containing protein 1 (SET-1) [21] (Figure 2). TCF/LEF serves as the DNA binding factor for the transcriptional activation complex, whereas BCL9/BCL9-2 acts to bridge other factors required to activate Wnt transcription. Upon β-catenin translocation to the nucleus, the chromatin associated with WREs is altered to favor activation of target genes via β-catenin-mediated recruitment of histone remodeling complexes. The histone acetyltransferases, CBP/p300, are thought to acetylate nucleosomes associated with WREs, thereby promoting gene transcription, although their mechanism of action is not well understood [20,22]. In addition, in some studies, CBP/p300 has been shown to inhibit the transcription of Wnt target genes, although this role may be context dependent [23]. Another chromatin remodeler, SET-1, a histone methyltransferase, adds methyl groups to lysine 4 of histone 3 (H3K4me), a mark associated with active transcription [21].

### 3.2. T-Cell Factor/Lymphoid Enhancer Factor

The TCF/LEF transcription factors are the major mediators of Wnt-responsive transcription. TCF/LEFs are highly conserved; most invertebrates have a single ortholog, whereas in most vertebrates they are encoded by four genes [24]. The four human members in this family include TCF1 (*TCF7* gene), TCF3 (*TCF7L1* gene), TCF4 (*TCF7L2* gene), and LEF1 [24]. TCF/LEF family members bind to WREs through their high mobility group (HMG) domain. TCF/LEF proteins have been shown to bind to the consensus sequence A/T A/T C A A A G in the minor groove, resulting in distortion of the DNA [25]. In addition, TCF/LEF proteins also act as transcriptional scaffolds to coordinate and position other factors involved in Wnt target gene regulation.

In *Drosophila*, there is a single copy of TCF, which exhibits both activator and repressor function [26]. However, there is increasing evidence suggesting activator and repressor functions of TCF are performed by separate TCF family members in vertebrates. For example, TCF3 is associated with repressor function, while LEF1 is associated with activator function, and, upon Wnt signaling in some contexts, there is a switch from TCF3 to LEF1 at Wnt target gene promoters. The role of TCF1 and TCF4 as either a repressor or activator of transcription remains unclear due to contradictions in different cell types and model systems [27,28].

### 3.3. Groucho/Transducing-Like Enhancer

Gro/TLE family members are transcriptional co-repressors that bind TCF/LEF [29,30]. Gro/TLE proteins form a homotetramer via their Q domain, which is important for their co-repressor activity [31,32]. Gro/TLE binds histone-modifying proteins, notably histone deacetylases, resulting in compaction of the chromatin and transcriptional silencing [33]. Furthermore, this function is dependent on their binding to TCF/LEF [34].

According to the prevailing model, the transition from constitutive transcriptional repression to Wnt/β-catenin-mediated activation is marked by the replacement of Gro/TLE by β-catenin on TCF/LEF. Early models suggest simple competition between β-catenin and TLE for binding to TCF/Lef [35]. Subsequent studies have indicated that dissociation of Gro/TLE may be facilitated [36,37].

The interaction between Gro/TLE and TCF/LEF may also be regulated by ubiquitination. The E3 ligase X-linked inhibitor of apoptosis (XIAP), upon phosphorylation by GSK3 in a Wnt-dependent manner, has been shown to ubiquitinate TLE, thereby decreasing its affinity for TCF/LEF [36,38]. Another E3 ubiquitin ligase, Hyd (hyperplastic discs)/UBR5, has also been proposed to ubiquitinate Gro/TLE [37]. It is unclear if both ligases function in the same tissues or if Gro/TLE ubiquitination is a tissue-specific process. Finally, based on the fact that certain vertebrate TCFs encode either repressor or activator function and that repressive TCF proteins have higher affinities for Gro/TLE, a model has been proposed by which Gro/TLE in complex with repressive TCFs (e.g., TCF3) is released upon Wnt signaling, allowing for the recruitment of activating TCFs (e.g., LEF1) bound to β-catenin [18].

### 3.4. Pygopus

Pygo, a Plant Homology Domain (PHD) domain protein, was identified as an essential Wingless (Wg)/Wnt signaling component in *Drosophila* [39,40,41,42]. Initial studies proposed that Pygo served to recruit and capture β-catenin to WREs via interaction with BCL9 [43]. Pygo has subsequently been shown to interact with the mediator complex subunits Med12 and Med13 to recruit general transcription factors to chromatin [44]. Another function attributed to Pygo is a role in chromatin remodeling. Pygo interacts with the histone acetyltransferase, CBP, as well as histone H3K4me3 (trimethylated lysine 4), a hallmark of active chromatin [45,46]. Interaction between Pygo and H3K4me3 has been proposed to involve the recruitment of Pygo by the core Chip/LDB-SSDP (ChiLS) Wnt enhanceosome complex (see below) [5].

The role of Pygo in Wnt signaling in mammals appears to be much more complicated than in *Drosophila*. In humans and mice, Pygo is encoded by two genes, *Pygo1* and *Pygo2*. Although *Pygo2* knockout mice die shortly after birth, they have a reduction in overall Wnt signaling and defects in some Wnt-dependent tissues (e.g., brain, eyes, hair follicles, and lungs) [47]. Surprisingly, intestinal development, which is strongly regulated by Wnt signaling, is not disrupted. In contrast, *Pygo1* knockout mice did not exhibit an observed phenotype [48]. Consistent with this, composite *Pygo1/2* double knockouts and *Pygo2* knockouts have similar phenotypes. Further evidence that Pygo may not be required for all Wnt signaling comes from a study demonstrating that Pygo-containing complexes promote transcription of certain Wnt target genes, but not others (e.g., genes that regulate cardiac tissue) [49]. Interestingly, the requirement for Pygo2 in mouse lens development is independent of β-catenin, suggesting a non-Wnt/β-catenin function for Pygo2 [47]. These, studies reveal that unlike its role in *Drosophila*, the role of Pygo in mammals is context-dependent and may function to modulate Wnt signaling responses rather than being an essential component.

### 3.5. B-cell CLL/Lymphoma 9

Similar to Pygo, early studies of Lgs/BCL9 were performed in *Drosophila*, which contain a single version of BCL9, Legless (Lgs) with 3 homology domains (HD1-3). The HD1 domain of Lgs was shown to bind to Pygo, whereas the HD2 domain was shown to bind directly to β-catenin. Although Lgs has an HD3 domain, it was found to be dispensable for Wingless signaling [40]. In mammals, there are two versions of BCL9, BCL9 and BCL9L (or BCL9-2), that are functionally redundant and contain five homology domains (HD1-5) [20]. Like that of Pygo, the role of BCL9 in Wnt signaling in mammals appears to be much more complicated than in *Drosophila*. In contrast with core Wnt pathway components, the specification of intestinal stem cells does not require BCL9. However, loss of BCL9 results in reduced expression of stem cell markers and reduced regeneration of the intestinal epithelium after injury [50]. Based on these studies, it was proposed that the primary function of Lgs/BCL9 is to link β-catenin to the WREs through its interaction with Pygo.

There appear to be differences in the function of BCL9 and Lgs in Wnt signaling between vertebrates and *Drosophila* as well. For example, the HD4 and HD5 domains of vertebrate BCL9, which are missing in Lgs, are required for Wnt transcriptional activation in mammalian cells [20]. Although the details may differ, similar to its *Drosophila* counterpart, mammalian BCL9 has been proposed to act as a dynamic scaffolding protein during Wnt signaling [51]. BCL9 interacts with TLE, possibly by facilitating the repositioning of transcriptional factors (e.g., TCF/LEF and ChiLS complex) to allow derepression of WREs and activation of transcription. Similarly, BCL9 interacts with histone remodeling proteins and, upon Wnt signaling, alters its interaction partner from histone deacetylases to the histone acetyltransferase, CBP [51]. BCL9 has also been identified as an oncogene in multiple myeloma where BCL9 is upregulated, and disrupting the β-catenin–BCL9 interaction has been shown to decrease transcription of Wnt target genes in colorectal cancer [52].

### 3.6. Non-TCF/LEF Transcription Partners of β-catenin

Hypoxia Inducible Factor (HIF)-1α controls cellular responses to low oxygen; in the absence of oxygen, HIF-1α is stabilized and acts as a transcription factor for hypoxia-responsive genes [53]. β-catenin binds HIF-1α [54]. Furthermore, this interaction has been shown to inhibit Wnt-dependent transcription to promote the transcription of genes required for neuronal differentiation [55,56].

A similar phenomenon has been observed with the Forkhead Box O (FOXO) proteins, which are involved in insulin signaling and in oxidative stress [57]. FOXO proteins translocate to the nucleus to facilitate transcription of target genes to directly compete with TCF/LEF for binding to β-catenin. Interaction between β-catenin and FOXO proteins induces the transcription of genes involved in cell cycle arrest as well as genes that prevent tissue injury [58,59,60].

Sox (Sry-related HMG box) transcription factors are another class of transcription factors that interact with β-catenin. Sox proteins play essential roles in animal development and in the maintenance of adult tissues [61]. Early evidence for Sox binding to β-catenin came from studies in Xenopus in which Sox17 and Sox3 were shown to compete with TCF/LEF for binding to β-catenin to inhibit Wnt signaling [62]. Studies in cultured cells demonstrated that Sox17 could also promote the degradation of TCF and β-catenin to block signaling [63]. Not all Sox proteins inhibit Wnt signaling; for example, Sox4 was shown to enhance TCF/β-catenin activity [63]. Consistent with their opposing action on the Wnt pathway, Sox17 and Sox4 are expressed in the normal gut epithelium in a mutually exclusive pattern [63].

## 4. Wnt/β-Catenin-Mediated Transcriptional Repression

Wnt/β-catenin signaling commonly results in conversion of a target gene from a repressed to an activated state. However, for certain genes, transcription is activated in the absence of Wnt signaling, and Wnt pathway activation represses gene transcription. In *Drosophila*, nearly 20% of all Wg-responsive genes are predicted to be repressed by Wg signaling [64]. Wnt-repressed genes have TCF/LEF binding sites, but these sites are distinct from the classical WREs. For these repressed genes, TCF promotes transcription via recruitment of coactivators in the absence of β-catenin; conversely, in the presence of β-catenin, the formation of a TCF-β–catenin complex represses transcription via recruitment of corepressors [65]. Many of the genes repressed by Wnt signaling are involved in other developmental signaling pathways. For example, during eyelid closure, Wnt signaling represses MAP3K1 expression, allowing for proper eye development [66]. Similarly, β-catenin functions to inhibit osteoblast differentiation to chondrocytes in the developing skeletal system by suppressing the expression of Runx2 and Sox9 [67]. Several other genes (*ADAM12*, *ITGB1*, *APP*, *DSP*, and *15-PGDH*) have also been shown to be directly inhibited by β-catenin in response to Wnt signaling [68,69,70].

## 5. Current Model: The Wnt Enhanceosome

By analogy to the large molecular size Wnt receptor signalosome at the plasma membrane and the β-catenin destruction complex in the cytoplasm, Fiedler et al. [5] coined the term “enhanceosome” to describe the nuclear complex that forms to drive Wnt-dependent gene transcription. At its core is the ChiLS complex, which is composed of two LDB (LIM-binding domain) and four SSDP (single-stranded DNA binding protein) proteins that bridge the Pygo protein to TCF/LEF via BCL9.

**Figure 3:**
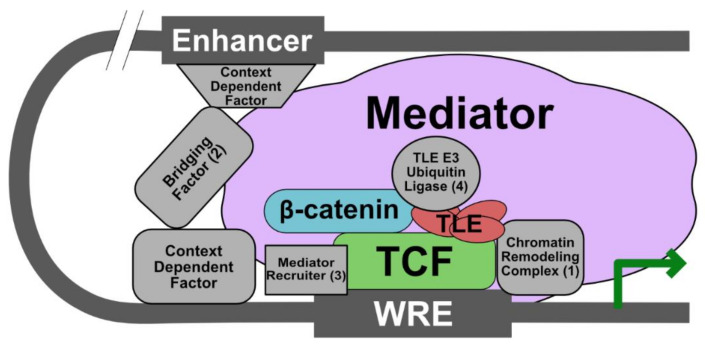
Proposed modified model of a Wnt enhanceosome with a β-catenin-TCF/LEF core complex and other requisite components that are context dependent. We propose that at least four additional components are required to form a functional enhanceosome: (1) a chromatin remodeling complex (e.g., SET-1, a histone methyltransferase, and CBP/p300, a histone acetyltransferase) that promotes gene transcription; (2) a bridging factor to link enhancer regions to the WRE and which may coordinate context-dependent factors (e.g., the ChILS complex and BCL9); (3) a mediator recruiter (e.g., Pygo and β-catenin); (4) an E3 ubiquitin ligase that promotes Gro/TLE dissociation from TCF/LEF (e.g., XIAP or Hyd/UBR5).

Unlike the signalosome and the β-catenin destruction complex, the universality of the ChiLS-enhanceosome model in Wnt signaling is unclear, given context dependent differences in enhanceosome components and the controversial role of Pygo as an essential component in vertebrate Wnt signaling [48,49]. This idea is consistent with knockout experiments showing that different phenotypes result from knockout of different genes involved in nuclear Wnt signaling, suggesting effects on distinct sets of genes between different nuclear components [1]. Evidence in *Drosophila* for a context-dependent enhanceosome comes from studies of Earthbound/Jerky and Erect wing (Ewg) [71]. Earthbound binds to the β-catenin/TCF complex to promote transcription in certain tissues and to activate a subset of Wg target genes. In addition, Earthbound is required for all Wnt-dependent consequences of Apc1 loss in the adult intestinal epithelium [72]. Similarly, Ewg, which forms a complex with Earthbound, also promotes transcription of a subset of Wg target genes [73]. Although ChiLS may not be a core component of all Wnt signaling events, the concept of a Wnt enhanceosome as originally described by Fiedler et al. [5] is nevertheless a useful concept to build upon (Figure 3).

Based on studies indicating that β-catenin and TCF/LEF are absolutely required for transcription of all Wg target genes in *Drosophila*, we propose that these proteins are the universal Wnt enhanceosome components [64]. In order to be functionally active, however, the enhanceosome must also contain (1) a chromatin remodeler (remarkably, nearly all Wnt nuclear components have been shown to interact directly with this class of proteins), (2) a protein to communicate between the enhancer and promoter regions of the Wnt target gene, (3) a protein that recruits Mediator complex components to promote general transcription (it is possible β-catenin may be sufficient to fulfill this role), and (4) a protein that regulates the interaction between TLE and TCF/LEF (i.e., an E3 ubiquitin ligase). In the future, it will be interesting to determine how the composition of the enhanceosome is dynamically altered in response to Wnt stimulation in different tissues and for crosstalk with other pathways.

## 6. Conclusions

Constitutive activation of Wnt signaling is associated with the development of numerous cancers [1]. A major role for Wnt pathway activation in the genesis of these cancers is highlighted by the fact that nearly 100% of all non-hereditary colorectal cancers exhibit inappropriate activation of the Wnt pathway, primarily due to mutations in the destruction complex (e.g., loss-of-function of APC/Axin or gain-of-function of β-catenin). To date, there are no FDA-approved Wnt inhibitors in clinical use for these diseases, and a drug that functions downstream of the major activating mutations would be particularly useful. Thus, a better understanding of nuclear events that drive Wnt signal transduction and the identification and characterization of new nuclear factors involved in Wnt signaling would lead not only to significant insights into the mechanisms by which a Wnt signal is propagated, but also to the development of drugs that target this pathway for the treatment of the majority of Wnt-driven cancers in humans. For example, the identification of Wnt contextual factors associated with specific cancer types may provide an opportunity to develop selective agents that target Wnt signaling in the cancer while minimizing inhibition of Wnt signaling in normal tissues. Alternatively, given the dynamic nature of the enhanceosome complex and the well-characterized plasticity of cancer cell pathways, targeting the β-catenin-TCF/LEF axis may be the best way to provide sustained inhibition of Wnt signaling.
